# An efficient route to *N*-alkylated 3,4-dihydroisoquinolinones with substituents at the 3-position†

**DOI:** 10.1039/c7ra13627g

**Published:** 2018-02-07

**Authors:** Aoi Tazawa, Junki Ando, Kohei Ishizawa, Isao Azumaya, Hidemasa Hikawa, Minoru Tanaka

**Affiliations:** Sohyaku Innovative Research Division, Mitsubishi Tanabe Pharma Corporation 1000 Kamoshida-cho, Aoba-ku Yokohama-shi Kanagawa 227-0033 Japan tanaka.minoru@mw.mt-pharma.co.jp; Faculty of Pharmaceutical Sciences, Toho University 2-2-1 Miyama Funabashi Chiba 274-8510 Japan isao.azumaya@phar.toho-u.ac.jp

## Abstract

A facile synthetic procedure for the production of *N*-alkylated 3,4-dihydroisoquinolinone derivatives is described. The desired products were obtained by *N*-alkylation of 3,3′-dimethyl-3,4-dihydroisoquinoline derivatives followed by oxidation of the resulting iminium salts. Reaction conditions for both steps were very mild and the desired cyclization products could be obtained in good yield. This strategy allows the generation of *N*-substituted 3,4-dihydroisoquinolinone derivatives with substituents at the 3-position.

## Abstract 2

*N*-Alkylated 3,4-dihydroisoquinolinone derivatives could be synthesized by *N*-alkylation of 3,3′-dimethyl-3,4-dihydroisoquinoline derivatives followed by oxidation of the resulting iminium salts.

## Graphical abstract

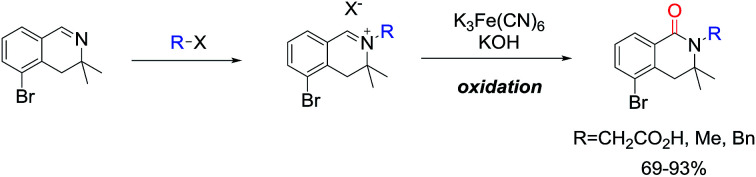

## Introduction

**Fig. 1:**
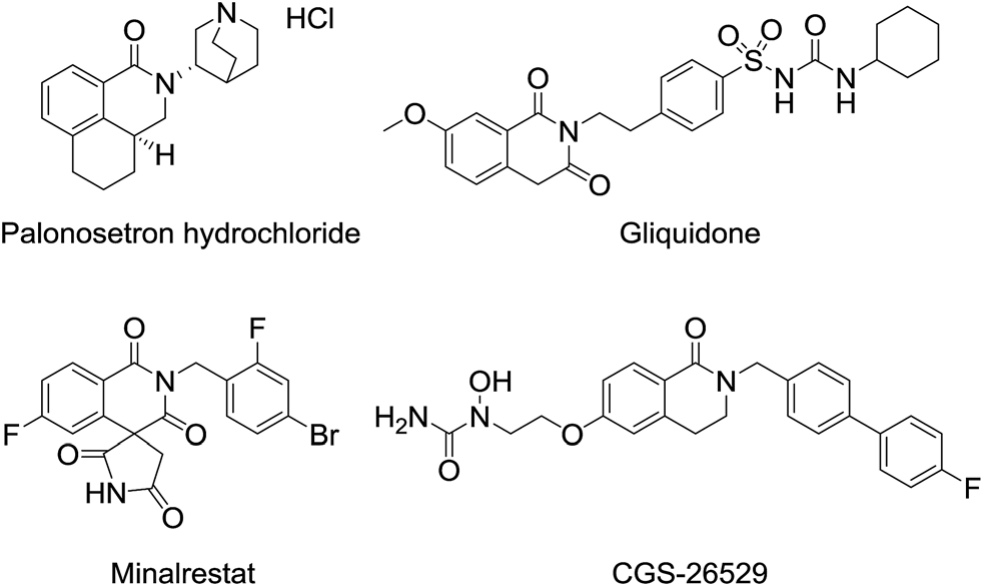
Representative pharmacologically active dihydroisoquinolinones.

3,4-Dihydroisoquinolinone 1 is an important member of the class containing a core structure found in compounds that exhibit biological and pharmacological properties, such as anti-nausea/vomiting,^1^ antidiabetic,^2,3^ and antiallergy/antiasthmatic^4^ activities. Some examples of pharmacologically active compounds that include the dihydroisoquinolinone moiety are shown in Fig. 1. Its suitable size and moderate polarity as a pharmacophore make 3,4-dihydroisoquinolinone a suitable scaffold that has been widely used in various drug candidates. For 3,4-dihydroisoquinolinone derivatives, the introduction of substituents at the 3-position generally improve their biostability^5^ because the substituent prevents oxidation of the unsubstituted 3-position. Nevertheless, only a few methods for effective preparation of *N*-alkylated 3,4-dihydroisoquinolinone with substituents at the 3-position are available, most of which are related to the synthesis of *N*-methyl analogues.^6^

**Scheme 1:**
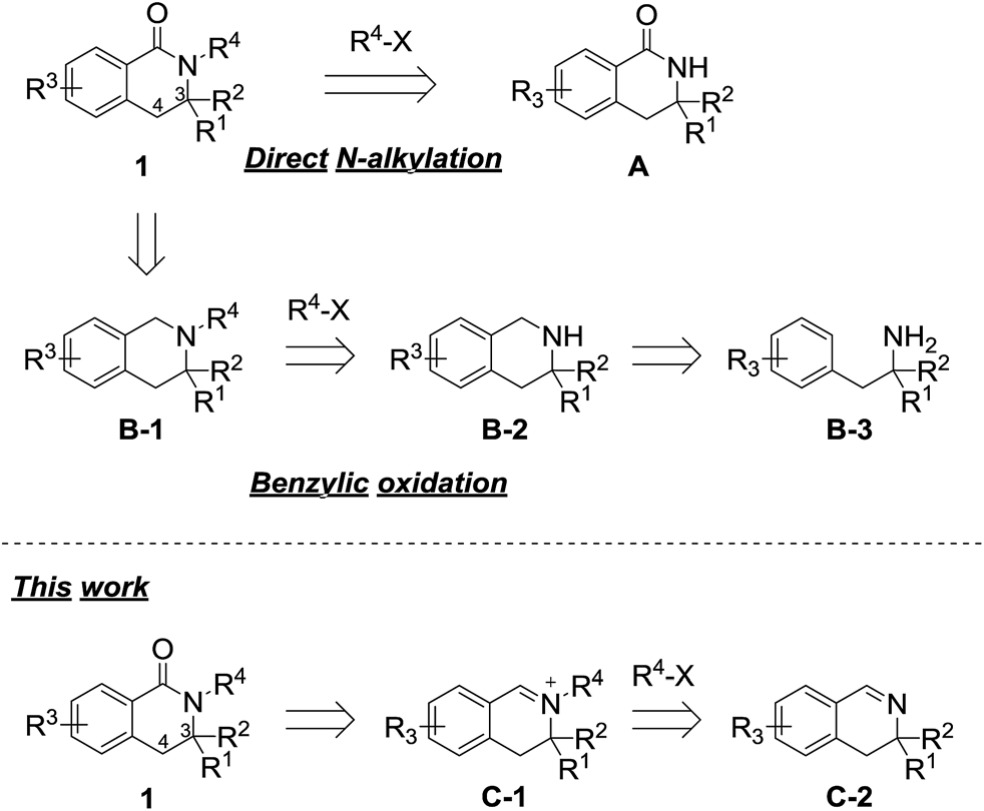
Retrosynthetic analysis of 3-substituted 3,4-dihydroisoquinolinones.

One of the simplest synthetic methods of the *N*-alkylated dihydroisoquinolinone skeleton is direct *N*-alkylation of *N*-unsubstituted dihydroisoquinolinone A (Scheme 1).^7^ The *N*-alkylation reaction, however, often does not proceed in the case of substrates with some substituents at the 3-position. Another method for preparing the skeleton involves benzylic oxidation of *N*-alkylated tetrahydroisoquinoline B-1.^8^ However, this route cannot be applied to substrates in which another position can be easily oxidized. Using this approach, the precursor is easy to synthesize because the nitrogen atom of tetrahydroisoquinolines B-2 is more nucleophilic than that of 3,4-dihydroisoquinolinones A. However, in the case of 3-substituted analogues, precursors B-2 are not obtained *via* cyclization reactions of the corresponding phenethylamine derivatives B-3.^9^

Thus, we have focused on the oxidation reaction of *N*-alkylated iminium salts C-1. As previous reports are limited to 3-unsubstituted analogues,^10^ we examined whether this strategy was compatible with 3-substituted dihydroisoquinolinone derivatives. In the course of the reaction, *N*-alkylation proceeds *via* a less sterically hindered transition state when compared with that of direct *N*-alkylation of dihydroisoquinolinone A. Therefore, this method has an apparent advantage for *N*-alkylation of sterically hindered analogues. In this work, we developed an effective synthetic method of 3-substituted *N*-alkyl-dihydroisoquinolinone derivatives *via* iminium intermediates.

## Results and discussion

### Preparation of 3,3-dimethyl-dihydroisoquinolinium salt

**Scheme 2:**
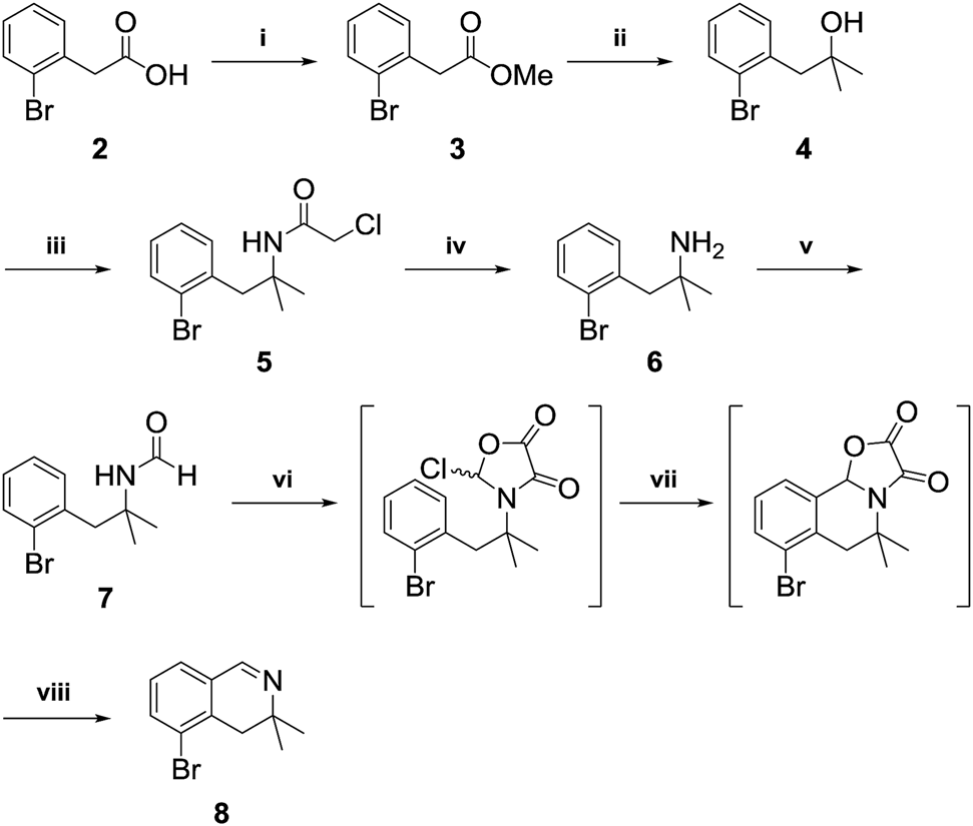
Reagents and conditions: (i) conc. H_2_SO_4_ (5 mol%), MeOH, reflux: (ii) MeMgBr (2.5 equiv.), THF, 0 °C to rt, 98% (2 steps); (iii) ClCH_2_CN (2 equiv.), conc. H_2_SO_4_ (1.3 equiv.), AcOH (3 equiv.), 0 °C to rt; (iv) thiourea (1.2 equiv.), EtOH–AcOH (5 : 1), 100 °C, quant (2 steps); (v) EtOCHO (2.0 M), 60 °C, 85%; (vi) (COCl)_2_ (1.1 equiv.), CH_2_Cl_2_; (vii) FeCl_3_ (1.5 equiv.); (viii) MeOH–H_2_SO_4_ (19 : 1), reflux, 89%.

3,3-Dimethyl-3,4-dihydroisoquinoline 8, was prepared by referring to a previously described procedure (Scheme 2).^11^ Our synthesis began with commercially available 2-bromo-phenylacetic acid 2, which was converted into an ester and reacted with methyl Grignard reagent to afford the tertiary alcohol 4. Nucleophilic substitution of 4 with chloroacetonitrile followed by reaction with thiourea afforded the tertiary amine 6^12^ in high yield. Amidation of the tertiary amine 6 with ethyl formate under neat conditions produced the formamide 7 in 85% yield. Tandem cyclization of 7 using oxalyl chloride followed by iron(iii) chloride afforded a tricyclic intermediate, which was pyrolyzed by MeOH–H_2_SO_4_ to give 3,3-dimethyl-3,4-dihydroisoquinoline in 89% yield.

**Scheme 3:**
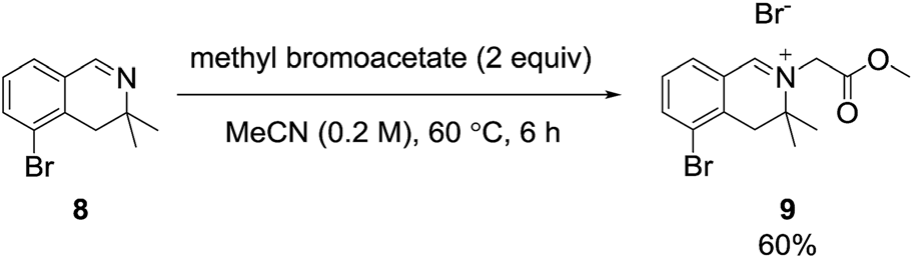
Alkylation of 3,3-dimethyl-3,4-dihydroisoquinoline.

After obtaining 3,3-dimethyl-3,4-dihydroisoquinoline 8, we examined the formation of isoquinolinium key precursors 9. For the preparation of the 3,3-dimethyl-dihydroisoquinolinium salt 9 (Scheme 3), dihydroisoquinoline 8 was reacted with methyl bromoacetate (2 equiv.) in acetonitrile at 60 °C. After 6 h, the desired product 9 was produced and precipitated as a colorless powder. The obtained powder was sufficiently pure for use in the next reaction without further purification.

### Synthesis of 3-substituted dihydroisoquinolinones

We next examined the oxidation of the 3,4-dihydro-isoquinolinium salt 9. The reaction was first examined in the presence of hydrochloric acid and dimethyl sulfoxide with reference to a previously described procedure (Table 1, entry 1),^10*a*^ but the desired product was not obtained. Next, we focused on optimizing the oxidation conditions. When 9 was reacted with *m*-chloroperoxybenzoic acid, dealkylation of 9 to dihydroisoquinoline 8 occurred predominantly (entry 2). Employing Dess–Martin periodinane as the oxidant, the reaction became messy and a complex product mixture that included 8 was obtained (entry 3). When oxone was employed as the oxidant, no reaction was observed (entry 4). However, when 9 was reacted with oxone in the presence of sodium bicarbonate, desired product 10 was obtained as the major product (entry 5). This suggested that basic conditions were preferable for 3-substituted dihydroisoquinolinones. As the yield was not satisfactory, further experiments were conducted. Treatment of 9 with potassium ferricyanide in the presence of potassium hydroxide gave the desired product as the hydrolyzed carboxylic acid 11 in 69% yield (entry 6).^13^ Dealkylated compound 8 was also obtained as a minor product.

**Table 1:** Optimization of the oxidation reaction

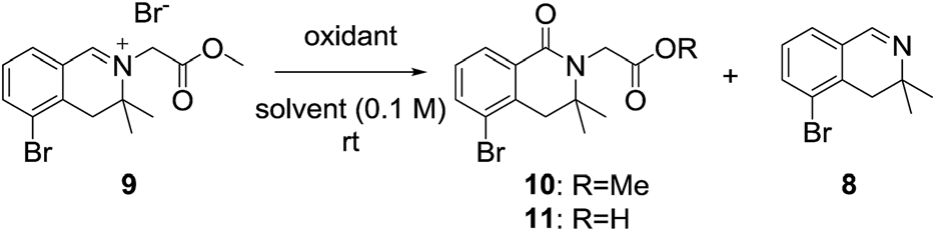		
Entry	Conditions^a^	Yields (%)
1	Conc. HCl, DMSO (1 : 7)	N.R.
2	*m*CPBA (1.5 equiv.), CH_2_Cl_2_	8: 88%
3	Dess–Martin periodinane (1.2 equiv.), CH_2_Cl_2_	Decomposed
4	Oxone (2 equiv.), MeCN/H_2_O (3 : 2)	N.R.
5	Oxone (2 equiv.), NaHCO_3_ (2 equiv.), MeCN/H_2_O (3 : 2)	10: 39%
6	K_3_Fe(CN)_6_ (6 equiv.), KOH (24 equiv.), dioxane/H_2_O (1 : 2)	11: 69%, 8: 25%

a 3,3-dimethyl-3,4-dihydroisoquinolinium salt 9 (0.1 mmol), oxidants, and solvent (0.1 M) were reacted for 12 h at ambient temperature.

**Scheme 4:**
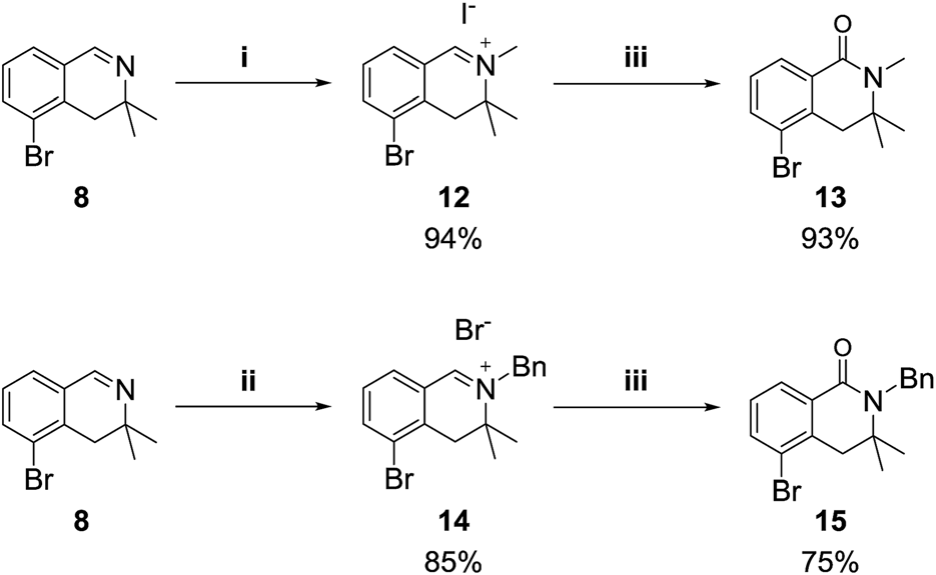
Reagents and conditions: (i) MeI (10 equiv.), acetone, rt; (ii) BnBr (10 equiv.), toluene, 70 °C; (iii) K_3_Fe(CN)_6_ (6 equiv.), KOH (24 equiv.), dioxane/H_2_O (1 : 2), rt.

**Scheme 5:**
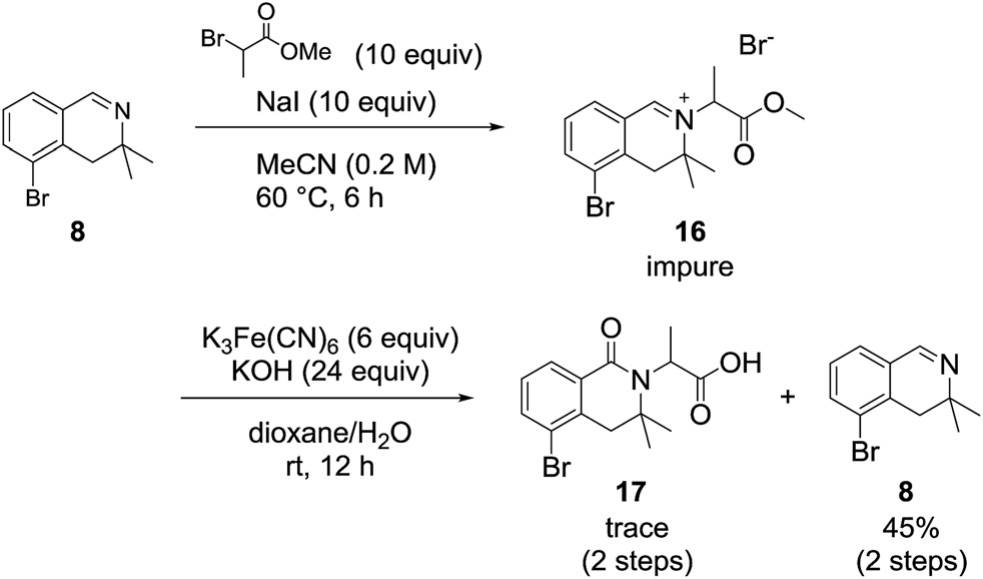
Oxidation of a 3,3-dimethyl-3,4-dihydroisoquino-linium derivative.

We also examined the oxidation reaction of isoquinolinium salts, including those with other alkyl substituents on the nitrogen atom.^14^ As expected, under optimal conditions, the desired dihydroisoquinolinones were obtained in high yields (Scheme 4). However, when dihydroisoquinolinium salt 16 was employed in the oxidation reaction, the desired product was obtained only in trace amounts, while dealkylated compound 8 was generated as the major product (Scheme 5). This was probably due to steric hindrance at the oxidation site.

### Investigation of the reaction mechanism

We hypothesize that the mechanism of the reaction involves a hydroxide ion as an oxygen source and proceeds through a radical pathway, because hexacyanoferrate(iii) is a typical one-electron oxidizing agent.^15^

**Scheme 6:**
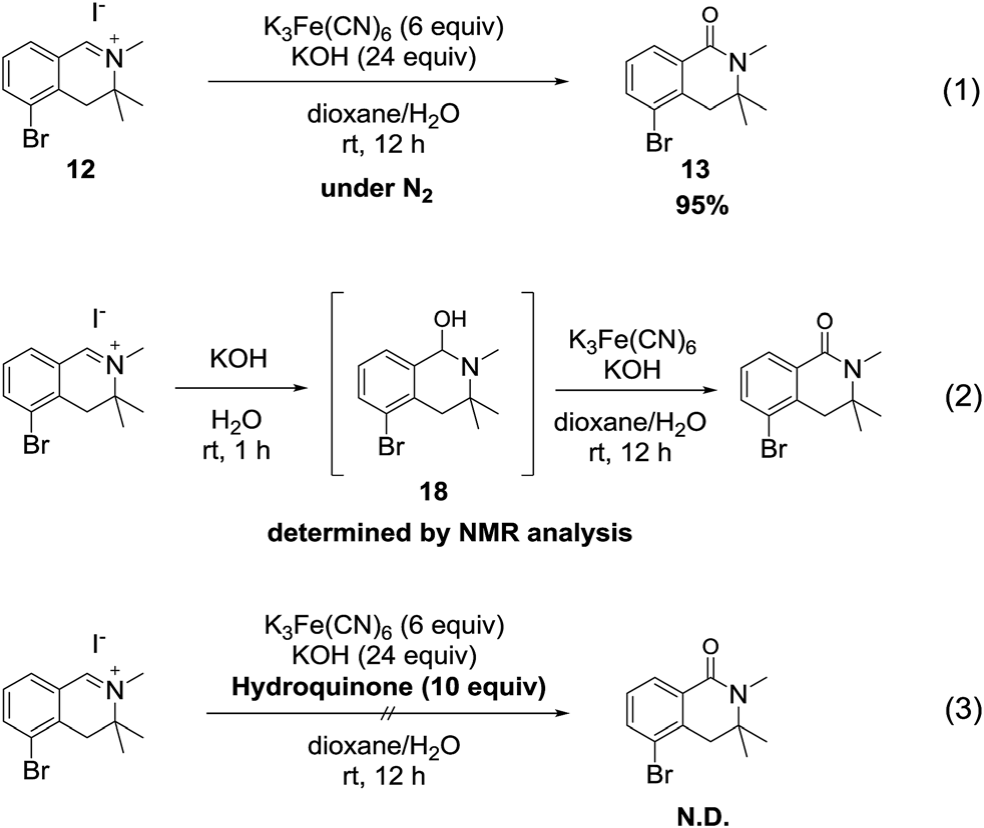
Control experiments for investigating the mechanism.

To verify this hypothesis, we conducted the oxidation reaction of isoquinolinium salt 12 under a nitrogen atmosphere. As a result, the reaction proceeded smoothly and the desired product 13 was obtained in excellent yield (Scheme 6, eqn (1)), suggesting that atmospheric oxygen (O_2_) was not the oxygen source. Next, we investigated the reaction of 12 with only potassium hydroxide solution to confirm that hydroxide ions were the oxygen source. The tetrahydroisoquinolinol intermediate 18 was determined by LC/MS and ^1^H NMR analysis^16^ of the crude mixture. Furthermore, when potassium ferricyanide was added to the solution of intermediate 18, generation of the desired product was detected (eqn (2)). When the reaction was conducted in the presence of a radical scavenger, the reaction mixture became messy and the desired product was not detected (eqn (3)). Based on these experimental results, we concluded that the oxidation reaction proceeded through a radical process *via* the tetrahydroisoquinolinol intermediate 18.

## Conclusions

We have developed a novel and efficient approach for the preparation of 3-substituted *N*-alkyl-3,4-dihydroisoquinolinones *via* the oxidation of iminium intermediates. Using this method, *N*-alkyl-3,4-dihydroisoquinolinones bearing substituents at the 3-position, which had not been obtained by *N*-alkylation of the amide nitrogen, could be obtained in moderate yield. We believe this method will contribute to the development of pharmacologically active compounds based on this skeleton. However, there is currently a limitation of the substituent patterns on the nitrogen atom such as a reactive methylene moiety, which we are currently trying to overcome by searching for various reaction conditions including oxidizing reagents.

## Experimental

### General information

^1^H and ^13^C NMR spectra were recorded on a Bruker 400 ULTRASHIELD PLUS. ^1^H and ^13^C chemical shifts are reported in ppm downfield from tetramethylsilane (TMS, *δ* scale) with the solvent resonances as internal standards. The following abbreviations were used to explain the multiplicities: s, singlet; d, doublet; t, triplet; q, quartet; m, multiplet; band, several overlapping signals; br, broad. IR spectra were recorded on a PerkinElmer Spectrum One FT-IR Spectrometer using attenuated total reflection (ATR). Melting points (mp) were recorded on a BÜCHI Melting Point B-545. Mass spectra were provided at the DMPK Research Laboratory, Mitsubishi Tanabe Pharma Corporation.

#### *N*-(1-(2-Bromophenyl)-2-methylpropan-2-yl)formamide (7)

In a flask, 1-(2-bromophenyl)-2-methylpropane-2-amine 6^13^ (2.0 g, 8.77 mmol) and ethylformate (1.0 M, 8.77 mL) were placed and the mixture was stirred at 60 °C. The reaction proceeded very slowly. After 5 d, ethylformate (1.0 M, 8.77 mL) was added and stirred for another 5 d at 60 °C. The disappearance of the starting material was confirmed by LC/MS. The reaction mixture was concentrated *in vacuo* to afford *N*-(1-(2-bromophenyl)-2-methylpropan-2-yl)formamide 7 (1.92 g, 7.49 mmol) in 85% yield. mp 63.6–63.7 °C; ^1^H NMR (400 MHz, CDCl_3_) *δ*_H_: 8.11–8.01 (1H, m), 7.60–7.56 (1H, m), 7.29–7.08 (3H, m), 5.73–5.29 (1H, m), 3.29–3.03 (2H, m), 1.43–1.40 (6H, m); ^13^C NMR (100 MHz, CDCl_3_) *δ*_C_: 162.6, 160.8, 137.3, 135.9, 133.5, 133.1, 132.5, 132.4, 128.8, 128.2, 127.3, 127.1, 126.1, 126.0, 77.2, 76.8, 55.4, 54.0, 48.2, 43.6, 28.6, 27.4; IR (ATR cm^−1^) *ν*_max_: 3293, 2970, 2858, 1659, 1540, 1466, 1382, 1258, 1017, 759, 705, 657; HRMS (ESI) [M + H]^+^ calculated for C_11_H_15_BrNO: 256.03315, found: 256.03357.

#### 5-Bromo-2-(2-methoxy-2-oxoethyl)-3,3-dimethyl-3,4-dihydro-isoquinolin-2-ium bromide (9)

Methyl bromoacetate (0.078 mL, 0.84 mmol, 1.0 equiv.) was added to a solution of 5-bromo-3,3-dimethyl-3,4-dihydroisoquinoline 8 (200 mg, 0.84 mmol) in acetonitrile (4.2 mL, 0.20 M). The resulting mixture was stirred for 6 h at 60 °C. As the reaction proceeded, a colorless powder precipitated. After the reaction, the precipitate was filtered and washed with ethyl acetate to afford methyl 5-bromo-2-(2-methoxy-2-oxoethyl)-3,3-dimethyl-3,4-dihydro-isoquinolin-2-ium bromide 9 (195.4 mg, 0.50 mmol) in 60% yield. mp 182.7–182.8 °C; ^1^H NMR (400 MHz, DMSO-*d*_6_) *δ*_H_: 9.35 (1H, s), 8.20 (1H, dd, *J* = 7.7, 1.0 Hz), 7.97 (1H, dd, *J* = 7.7, 1.0 Hz), 7.59 (1H, dd, *J* = 7.7, 7.7 Hz), 5.19 (2H, s), 3.82 (3H, s), 3.35 (2H, s), 1.46 (6H, s); ^13^C NMR (100 MHz, DMSO-*d*_6_) *δ*_C_: 171.3, 167.7, 142.5, 136.6, 134.8, 130.7, 126.0, 124.1, 64.1, 64.1, 55.0, 53.8, 53.8, 23.7, 23.7; IR (ATR cm^−1^) *ν*_max_: 2925, 1748, 1644, 1417, 1223, 1198, 1129, 795, 693; HRMS (ESI) [M − Br]^+^ calculated for C_14_H_17_BrNO_2_: 310.04372, found: 310.04402.

#### 5-Bromo-2,3,3-trimethyl-3,4-dihydroisoquinolin-2-ium iodide (12)

5-Bromo-2,3,3-trimethyl-3,4-dihydroisoquinolin-2-ium iodide 12 was obtained following the same procedure described in detail for the preparation of isoquinolinium 9 from 8. mp 182.5–182.6 °C; ^1^H NMR (400 MHz, DMSO-*d*_6_) *δ*_H_: 9.24 (1H, s), 8.11 (1H, dd, *J* = 8.2, 1.0 Hz), 7.87 (1H, dd, *J* = 7.7, 1.0 Hz), 7.55 (1H, dd, *J* = 8.2, 7.7 Hz), 3.74 (3H, s), 3.28 (2H, s), 1.47 (6H, s); ^13^C NMR (100 MHz, DMSO-*d*_6_) *δ*_C_: 167.1, 141.2, 136.3, 133.5, 130.4, 126.5, 123.8, 62.6, 62.6, 42.7, 23.8, 23.8; IR (ATR cm^−1^) *ν*_max_: 2969, 1650, 1562, 1445, 1376, 1231, 1204, 1120, 901, 785, 700, 571; HRMS (ESI) [M − I^−^] calculated for C_12_H_15_BrN: 252.03824, found: 252.03869.

#### 2-Benzyl-5-bromo-3,3-dimethyl-3,4-dihydroisoquinolin-2-ium bromide (14)

2-Benzyl-5-bromo-3,3-dimethyl-3,4-dihydro-isoquinolin-2-ium 14 was obtained following the same procedure detailed for the preparation of isoquinolinium 9 from 8. mp 179.3–179.4 °C; ^1^H NMR (400 MHz, DMSO-*d*_6_) *δ*_H_: 9.39 (1H, s), 8.15 (1H, dd, *J* = 8.2, 1.0 Hz), 8.01 (1H, dd, *J* = 7.7, 1.0 Hz), 7.59–7.42 (5H, m), 5.38 (2H, s), 3.32 (2H, s), 1.42 (6H, s); ^13^C NMR (100 MHz, DMSO-*d*_6_) *δ*_C_: 168.6, 141.6, 136.6, 134.5, 133.5, 130.4, 129.6, 129.6, 129.4, 129.0, 129.0, 126.7, 123.7, 64.3, 64.3, 58.3, 24.6, 24.6; IR (ATR cm^−1^) *ν*_max_: 3313, 2921, 1642, 1561, 1448, 1375, 1254, 1194, 1129, 955, 789, 761, 700, 692; HRMS (ESI) [M − Br^−^] calculated for C_18_H_19_BrN: 328.06954, found: 328.06984.

#### 2-(5-Bromo-3,3-dimethyl-1-oxo-3,4-dihydroisoquinolin-2(1*H*)-yl)acetic acid (11)

Potassium hydroxide (8.0 M in water, 0.307 mL, 2.46 mmol, 24 equiv.) was added to a solution of potassium ferricyanide (137.7 mg, 0.614 mmol, 6.0 equiv.) in H_2_O (1.4 mL). After the reagent was dissolved, a solution of methyl 5-bromo-2-(2-methoxy-2-oxoethyl)-3,3-dimethyl-3,4-dihydro-isoquinolin-2-ium bromide 9 (40.0 mg, 0.102 mmol) in dioxane (0.7 mL) was added and the reaction mixture was stirred for 12 h at room temperature. The reaction mixture was washed with CHCl_3_ and the aqueous layer acidified to pH 4 by adding a 1 M hydrochloric acid solution. The aqueous layer was extracted with CHCl_3_, dried over Na_2_SO_4_ and concentrated to afford 2-(5-bromo-3,3-dimethyl-1-oxo-3,4-dihydroisoquinolin-2(1*H*)-yl)acetic acid 11 (22 mg, 0.070 mmol) in 69% yield. mp 169.4–169.5 °C; ^1^H NMR (400 MHz, CDCl_3_) *δ*_H_: 8.03 (1H, d, *J* = 7.7 Hz), 7.70 (1H, d, *J* = 8.2 Hz), 7.22 (1H, dd, *J* = 8.2, 7.7 Hz), 5.16 (1H, brs), 4.30 (2H, s), 3.11 (2H, s), 1.36 (6H, s); ^13^C NMR (100 MHz, CDCl_3_) *δ*_C_: 172.7, 164.9, 136.3, 136.0, 129.7, 128.3, 127.7, 123.1, 56.7, 44.2, 42.0, 26.8, 26.8; IR (ATR cm^−1^) *ν*_max_: 2967, 1703, 1644, 1467, 1402, 1329, 1258, 1168, 1123, 915, 808, 752, 653; HRMS (ESI) [M + H]^+^ calculated for C_13_H_15_BrNO_3_: 312.02298, found: 312.02296.

#### Methyl 2-(5-bromo-3,3-dimethyl-1-oxo-3,4-dihydroisoquino-lin-2(1*H*)-yl)acetate (10)

Sodium hydrogen carbonate (128.9 mg, 0.820 mmol, 4.0 equiv.) was added to a solution of Oxone® (943.2 mg, 0.820 mmol, 4.0 equiv.) in H_2_O (2 mL). Then, a solution of methyl 5-bromo-2-(2-methoxy-2-oxoethyl)-3,3-dimethyl-3,4-dihydroisoquinolin-2-ium bromide 9 (150 mg, 0.384 mmol) in acetonitrile (4.0 mL) was added and the reaction mixture was stirred for 12 h at room temperature. The reaction mixture was diluted with water and CHCl_3_. The aqueous layer was extracted with CHCl_3_, dried over Na_2_SO_4_ and concentrated *in vacuo*. The residue was purified by column chromatography (hexane : EtOAc = 3 : 1) to give methyl 2-(5-bromo-3,3-dimethyl-1-oxo-3,4-dihydroisoquinolin-2(1*H*)-yl)acetate 10 (48.2 mg, 0.148 mmol) in 39% yield. ^1^H NMR (400 MHz, CDCl_3_) *δ*_H_: 8.04 (1H, dd, *J* = 7.7, 1.5 Hz), 7.69 (1H, dd, *J* = 8.2, 1.0 Hz), 7.22 (1H, dd, *J* = 8.2, 7.7 Hz), 4.30 (2H, s), 3.76 (3H, s), 3.12 (2H, s), 1.35 (6H, s); ^13^C NMR (100 MHz, CDCl_3_) *δ*_C_: 170.4, 163.9, 136.1, 135.9, 130.1, 128.2, 127.7, 123.0, 56.2, 52.3, 43.4, 42.2, 26.9, 26.9; IR (ATR cm^−1^) *ν*_max_: 2950, 1750, 1643, 1562, 1455, 1413, 1386, 1199, 1167, 1012, 970, 754, 653; HRMS (ESI) [M + H]^+^ calculated for C_14_H_17_BrNO_3_: 326.03863, found: 326.03908.

#### 5-Bromo-2,3,3-trimethyl-3,4-dihydroisoquinolin-1(2*H*)-one (13)

5-Bromo-2,3,3-trimethyl-3,4-dihydroisoquinolin-1(2*H*)-one 13 was obtained following the same procedure described in detail for the preparation of isoquinolinium 11 from 9. mp 91.5–91.6 °C; ^1^H NMR (400 MHz, CDCl_3_) *δ*_H_: 8.06 (1H, dd, *J* = 7.7, 1.0 Hz), 7.66 (1H, dd, *J* = 8.2, 1.0 Hz), 7.21 (1H, dd, *J* = 8.2, 7.7 Hz), 3.09 (3H, s), 3.03 (2H, s), 1.33 (6H, s); ^13^C NMR (100 MHz, CDCl_3_) *δ*_C_: 163.7, 135.8, 135.4, 130.6, 128.1, 127.7, 122.9, 55.5, 41.9, 27.3, 26.3, 26.3; IR (ATR cm^−1^) *ν*_max_: 2967, 1636, 1566, 1455, 1409, 1369, 1163, 1101, 1042, 800, 732; HRMS (ESI) [M + H]^+^ calculated for C_12_H_15_BrNO: 268.03315, found: 268.03329.

#### 2-Benzyl-5-bromo-3,3-dimethyl-3,4-dihydroisoquinolin-1(2*H*)-one (15)

2-Benzyl-5-bromo-3,3-dimethyl-3,4-dihydroisoquinolin-1(2*H*)-one 15 was obtained following the same procedure described in detail for the preparation of isoquinolinium 11 from 9. mp 133.3–133.4 °C; ^1^H NMR (400 MHz, CDCl_3_) *δ*_H_: 8.13 (1H, dd, *J* = 7.7, 1.0 Hz), 7.69 (1H, dd, *J* = 8.2, 1.0 Hz), 7.33–7.20 (6H, m), 4.84 (2H, s), 3.06 (2H, s), 1.28 (6H, s); ^13^C NMR (100 MHz, CDCl_3_) *δ*_C_: 164.3, 139.5, 136.1, 135.7, 130.7, 128.5, 128.5, 128.2, 127.9, 127.1, 127.1, 126.8, 123.0, 56.7, 45.3, 42.7, 27.1, 27.1; IR (ATR cm^−1^) *ν*_max_: 2975, 1624, 1561, 1454, 1403, 1356, 1287, 1197, 1120, 990, 800, 744, 698, 666; HRMS (ESI) [M + H]^+^ calculated for C_18_H_19_BrNO: 344.06445, found: 344.06422.

## Conflicts of interest

There are no conflicts to declare.

## Supplementary Material

RA-008-C7RA13627G-s001
