# Supplementary material for: An efficient route to N-alkylated 3,4-dihydroisoquinolinones with substituents at the 3-position
Source: RSC Adv. 2018 Feb 7;8(11):6146–51. doi: 10.1039/c7ra13627g (PMC9078240; doi:10.1039/c7ra13627g)

Electronic Supplementary Material (ESI) for RSC Advances.
This journal is © The Royal Society of Chemistry 2018

Supplementary Data

An efficient route to N-alkylated 3,4-dihydroisoquinolinones with substituents at the 3-position

Aoi Tazawa,[ab] Junki Ando,[a] Kohei Ishizawa,[a]
Isao Azumaya,*[b] Hidemasa Hikawa[b] and Minoru Tanaka,*[a]

*[a] Sohyaku Innovative Research Division, Mitsubishi Tanabe Pharma Corporation, 1000 Kamoshida-cho, Aoba-ku, Yokohama-shi, Kanagawa 227-0033, Japan

Tel: +81-45-963-7217; fax: +81-45-963-7247

E-mail address: tanaka.minoru@mw.mt-pharma.co.jp

*[b] Faculty of Pharmaceutical Sciences, Toho University, 2-2-1 Miyama, Funabashi, Chiba 274-8510, Japan

Tel: +81-47-472-1589; fax: +81-47-472-1595

E-mail address: isao.azumaya@phar.toho-u.ac.jp

Table of Contents.

S2 General Information

S3-S11 NMR Spectra of compounds

General Information. ^{1}H and ^{13}C NMR spectra were recorded on a Bruker 400 ULTRASHIELD PLUS. ^{1}H and ^{13}C chemical shifts are reported in ppm downfield from tetramethylsilane (TMS, δ scale) with the solvent resonances as internal standards. The following abbreviations were used to explain the multiplicities: s, singlet; d, doublet; t, triplet; q, quartet; m, multiplet; band, several overlapping signals; br, broad. IR spectra were recorded on a PerkinElmer Spectrum One FT-IR Spectrometer using attenuated total reflection (ATR). Melting points (m.p.) were recorded on a BÜCHI Melting Point B-545. Mass spectra were provided at the DMPK Research Laboratory, Mitsubishi Tanabe Pharma Corporation.

N-(1-(2-Bromophenyl)-2-methylpropan-2-yl)formamide (7).

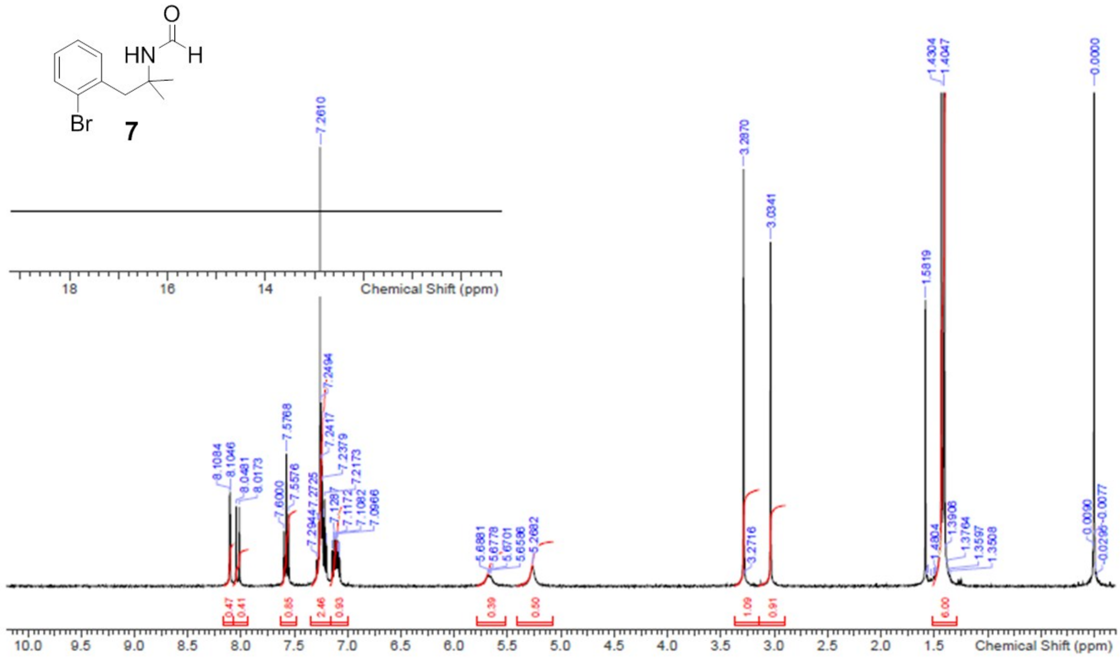

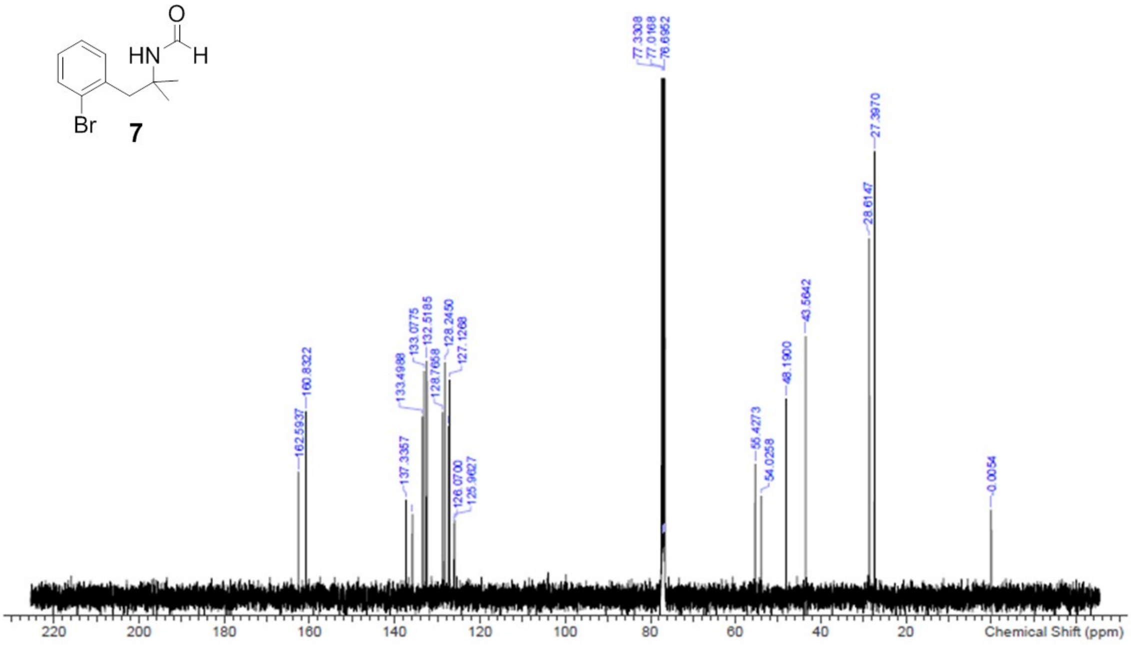

5-Bromo-2-(2-methoxy-2-oxoethyl)-3,3-dimethyl-3,4-dihydro-isoquinolin-2-ium bromide (9).

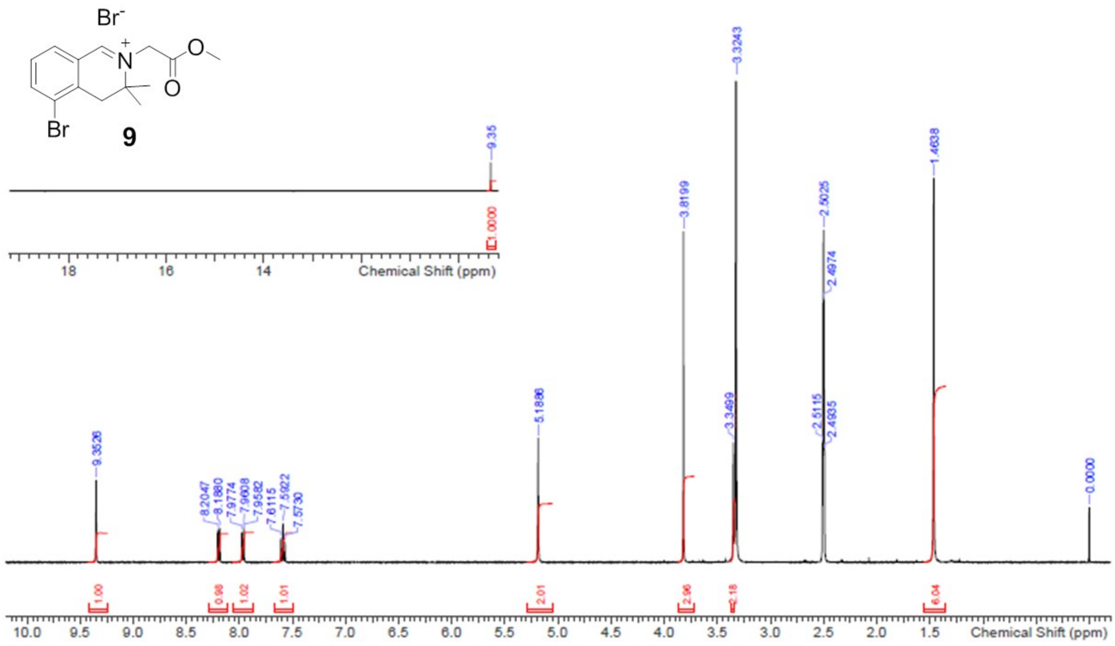

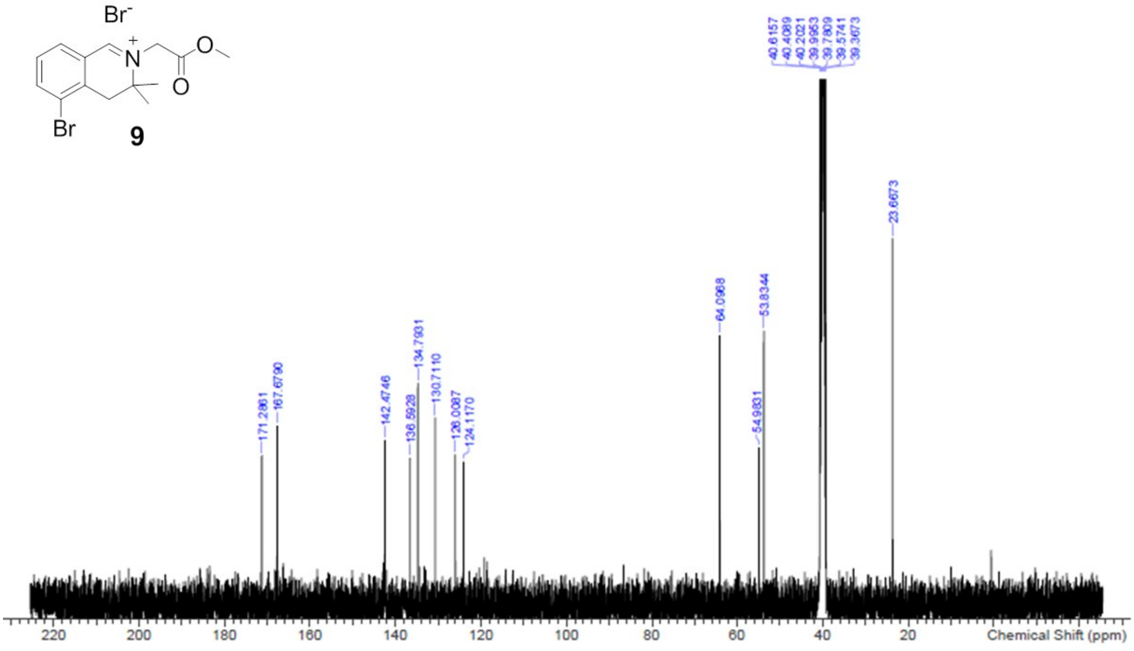

Methyl 2-(5-bromo-3,3-dimethyl-1-oxo-3,4-dihydroisoquino-lin-2(1*H*)-yl)acetate (10).

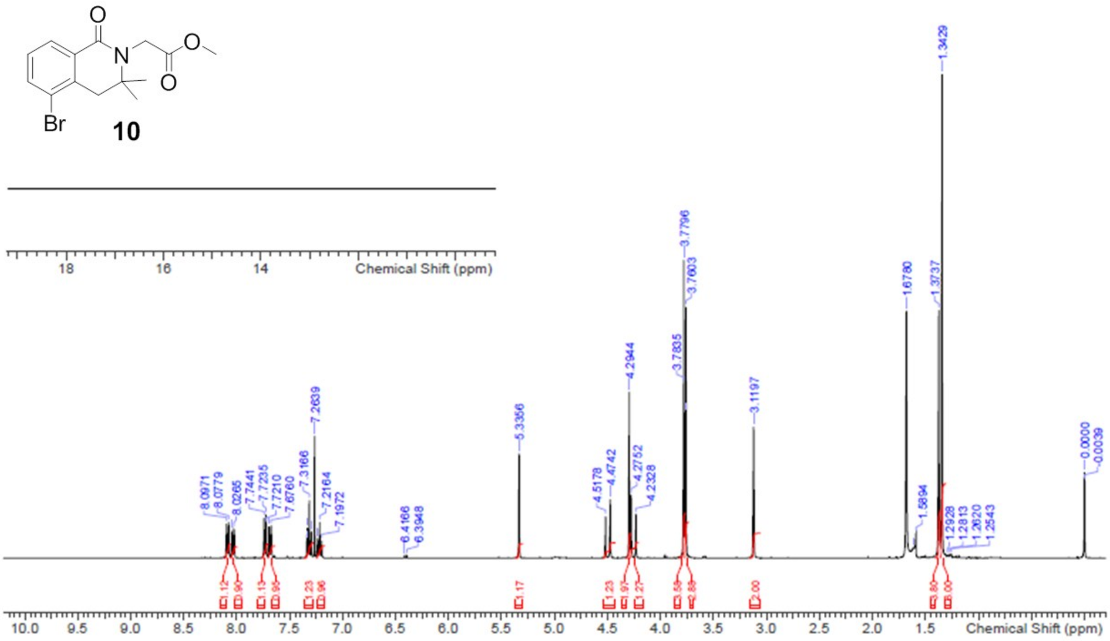

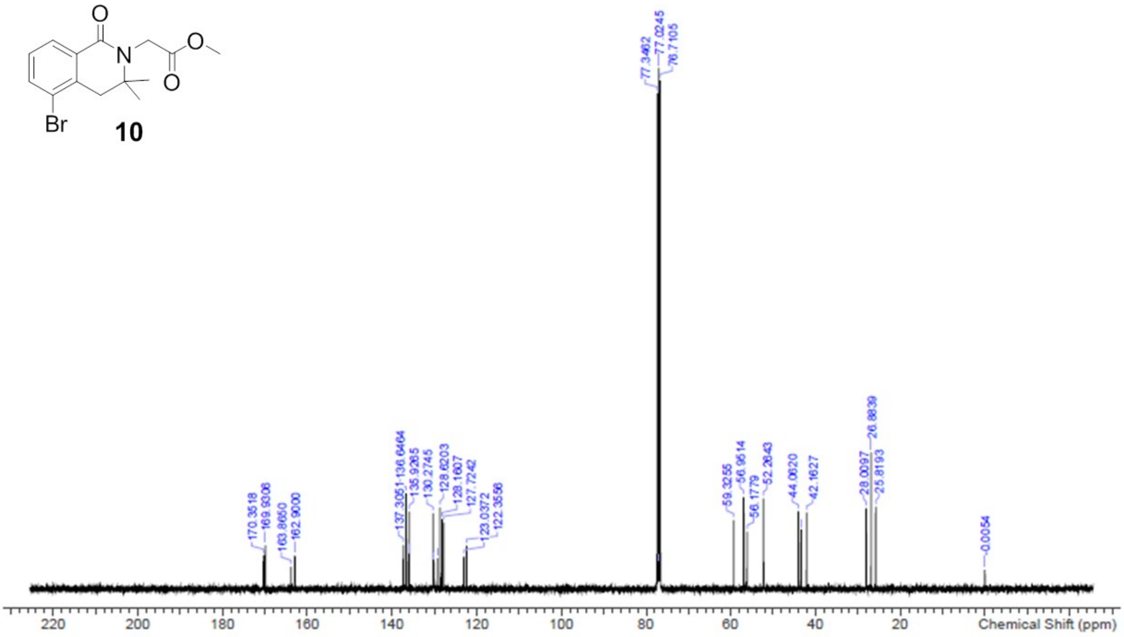

2-(5-Bromo-3,3-dimethyl-1-oxo-3,4-dihydroisoquinolin-2(1H)-yl)acetic acid (11).

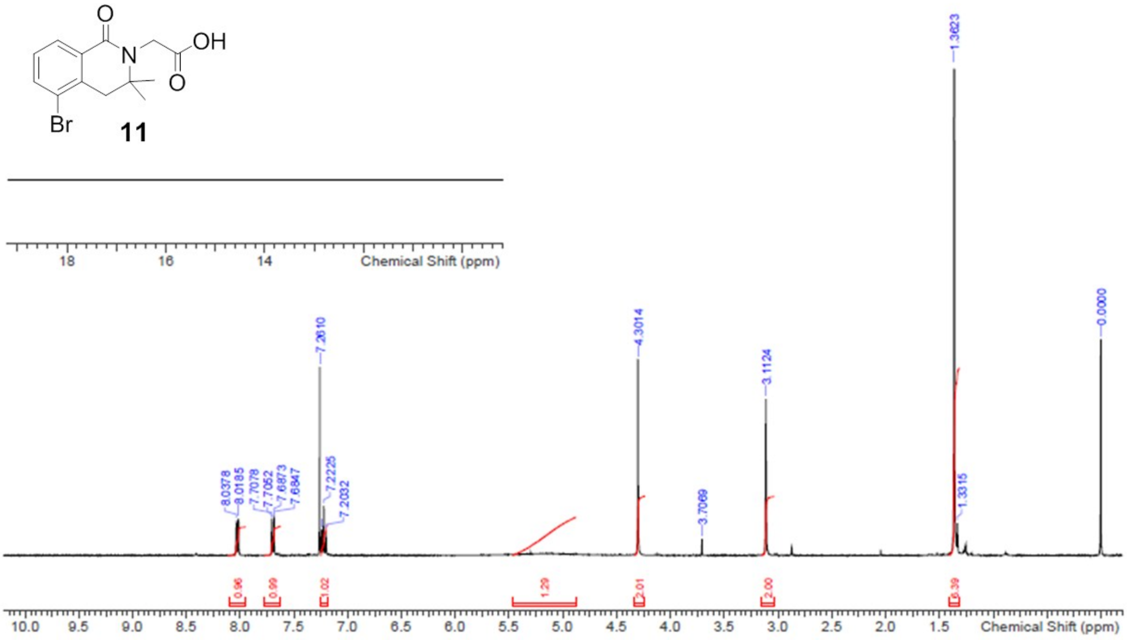

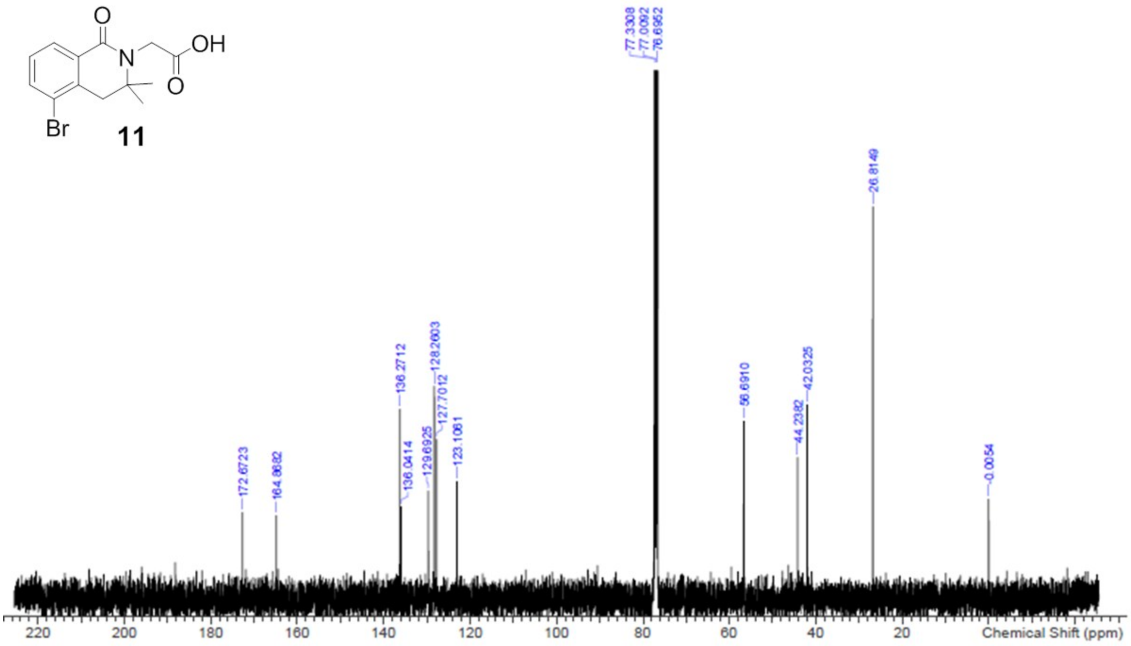

5-Bromo-2,3,3-trimethyl-3,4-dihydroisoquinolin-2-ium iodide (12).

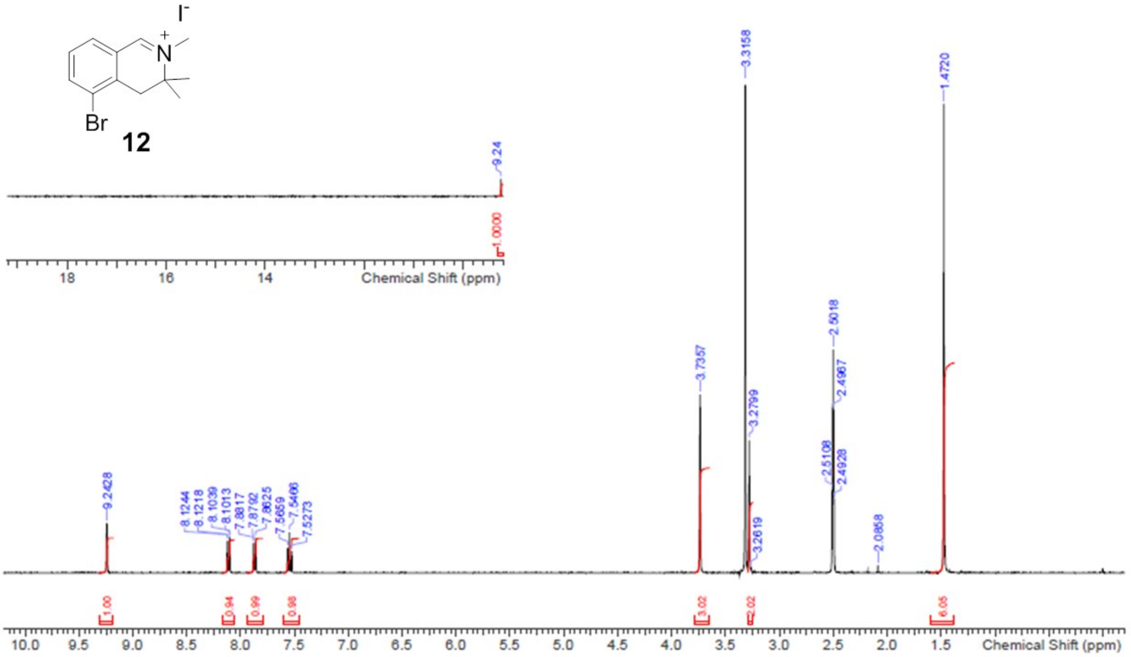

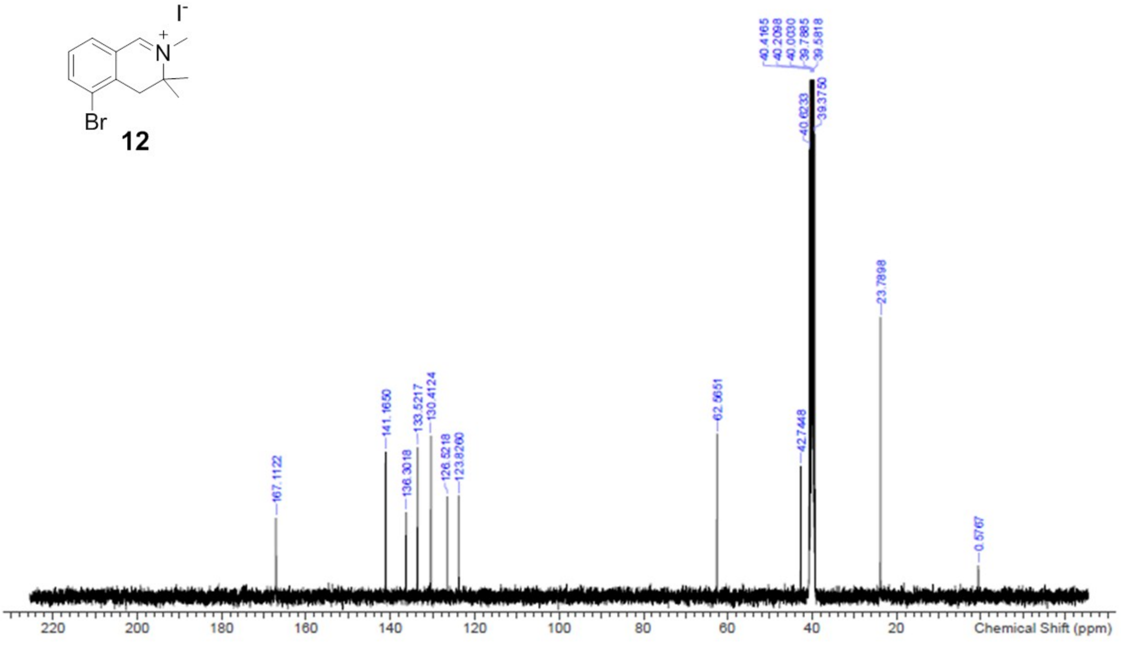

5-Bromo-2,3,3-trimethyl-3,4-dihydroisoquinolin-1(2*H*)-one (13).

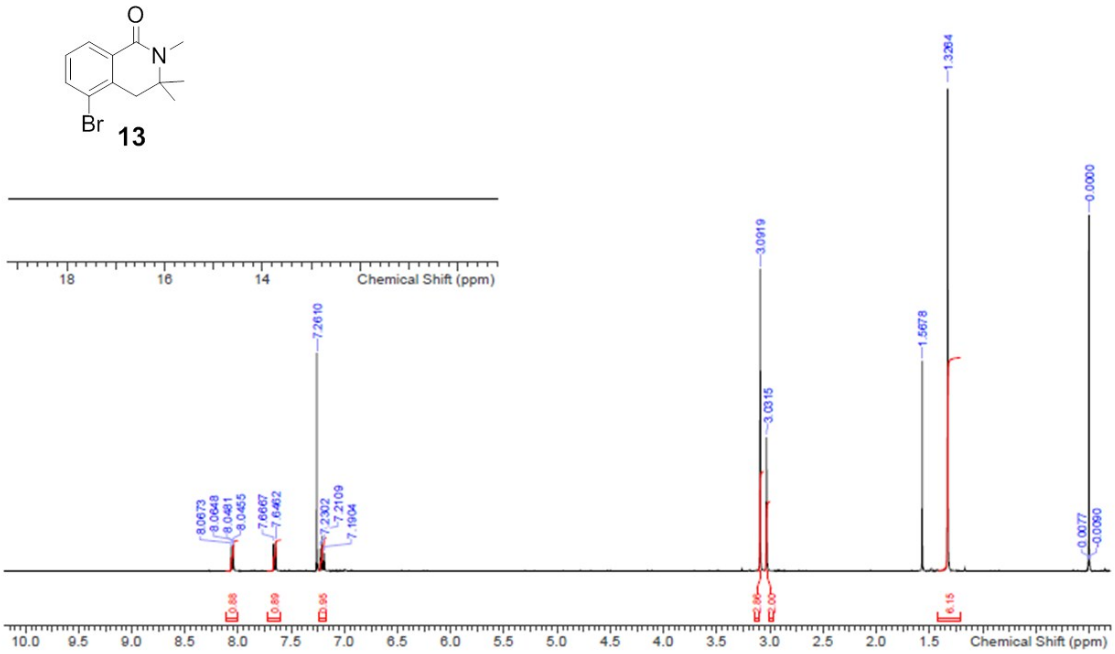

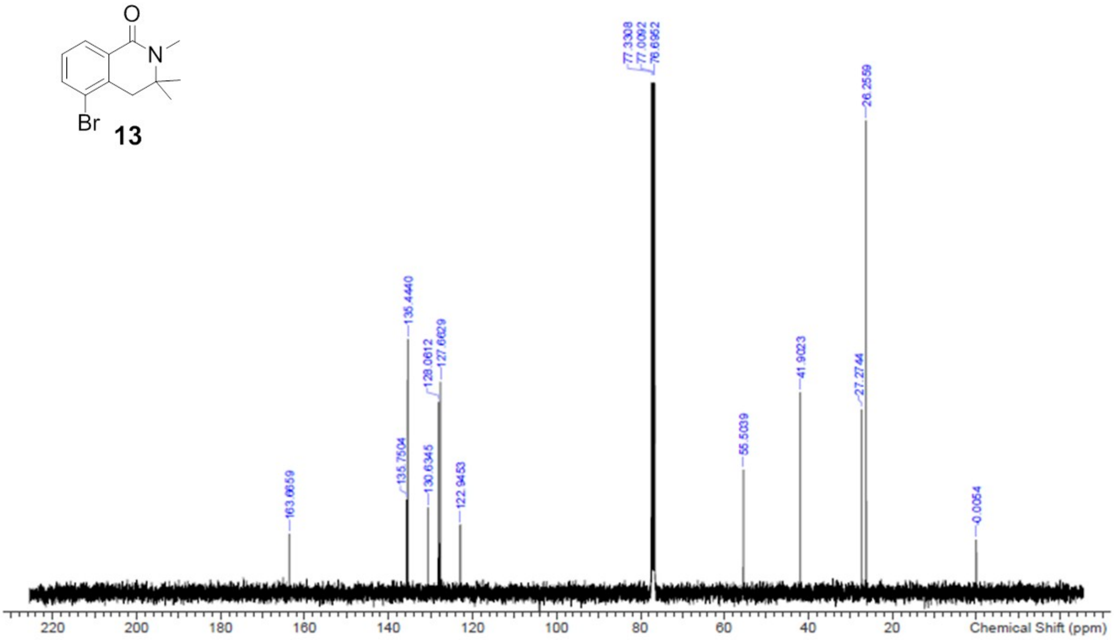

2-Benzyl-5-bromo-3,3-dimethyl-3,4-dihydroisoquinolin-2-ium bromide (14).

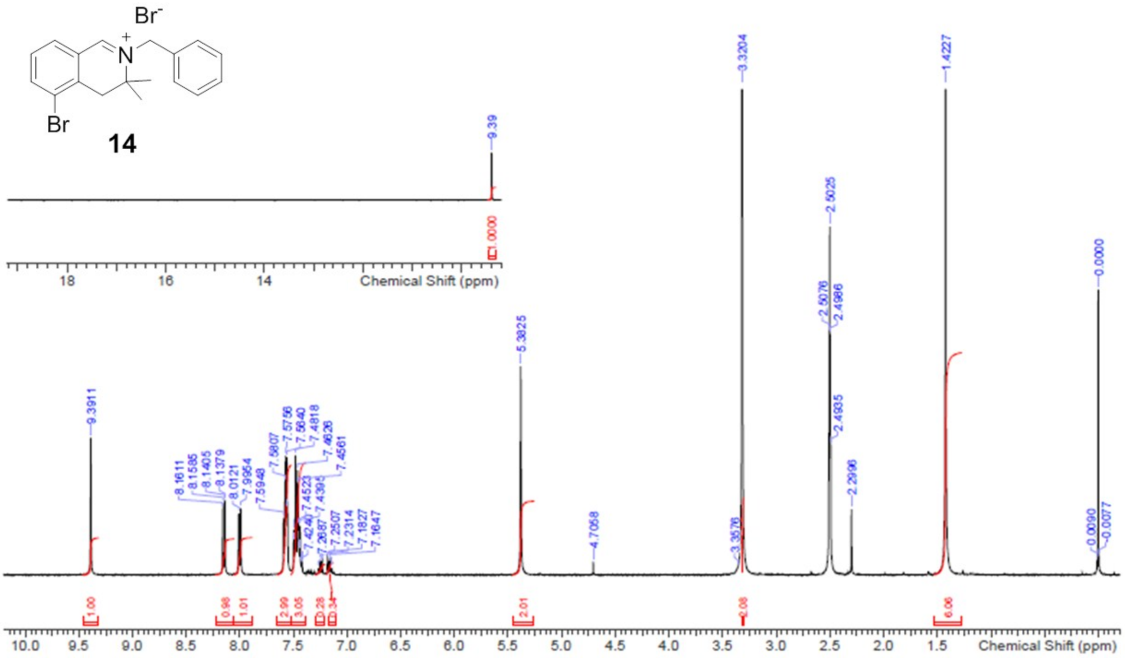

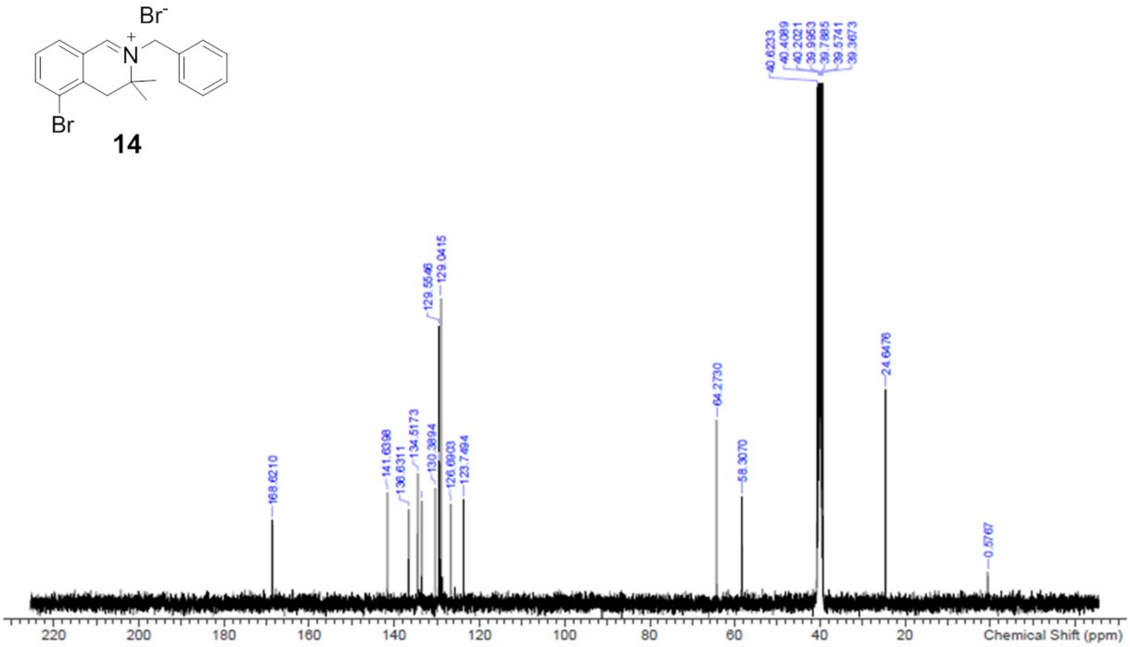

2-Benzyl-5-bromo-3,3-dimethyl-3,4-dihydroisoquinolin-1(2*H*)-one (15).

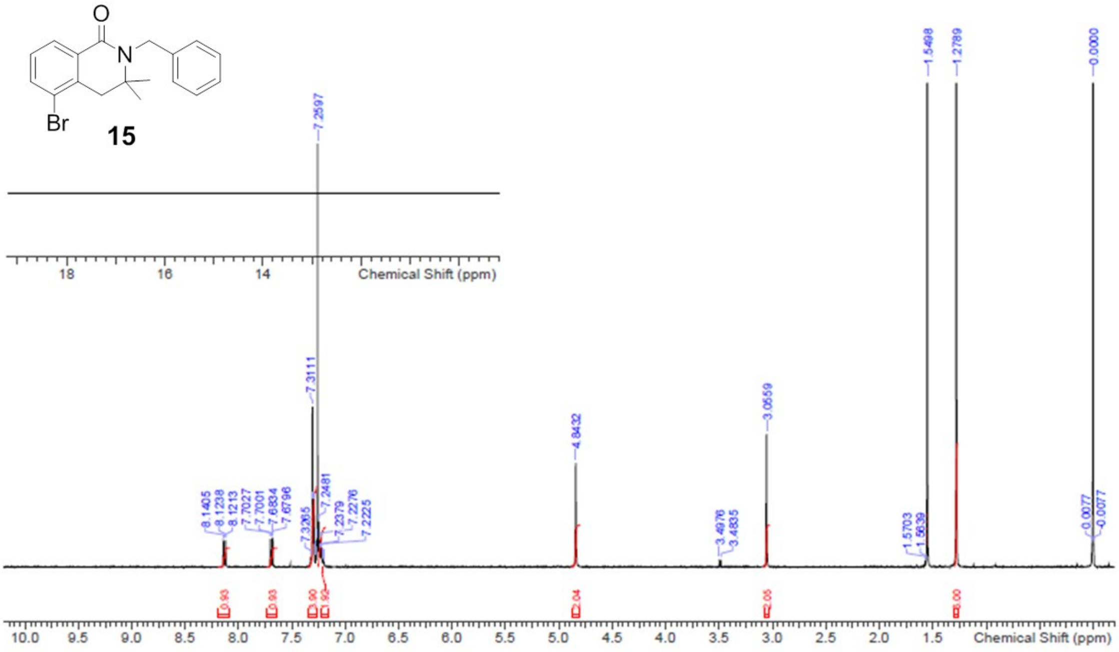

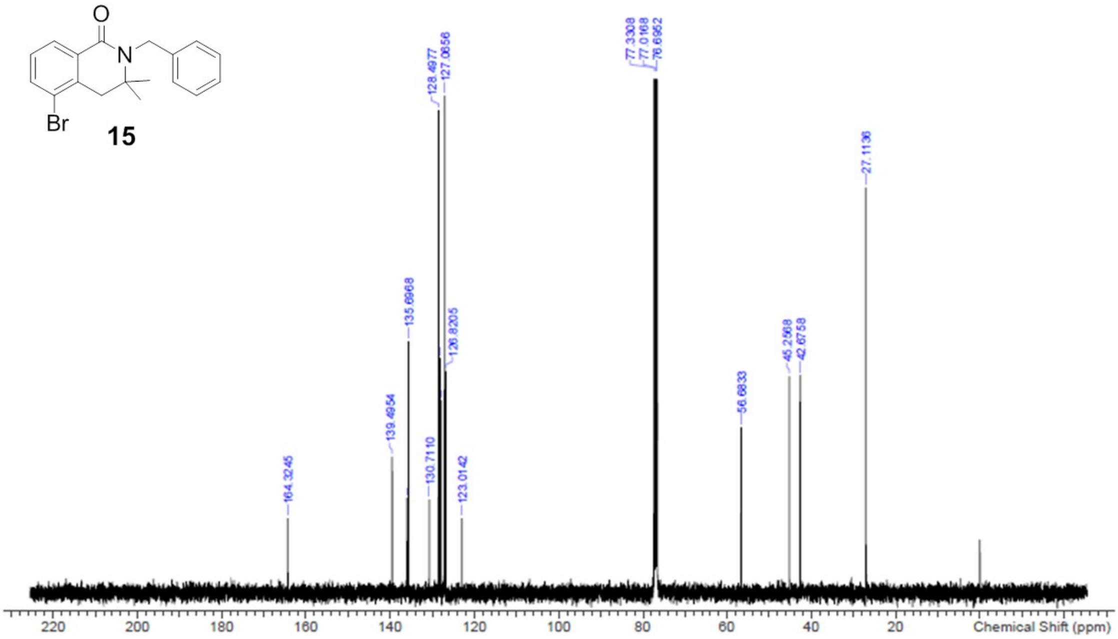

1,2,3,4-tetrahydro-2-methyl-isoquinolinol intermediate(**18).**

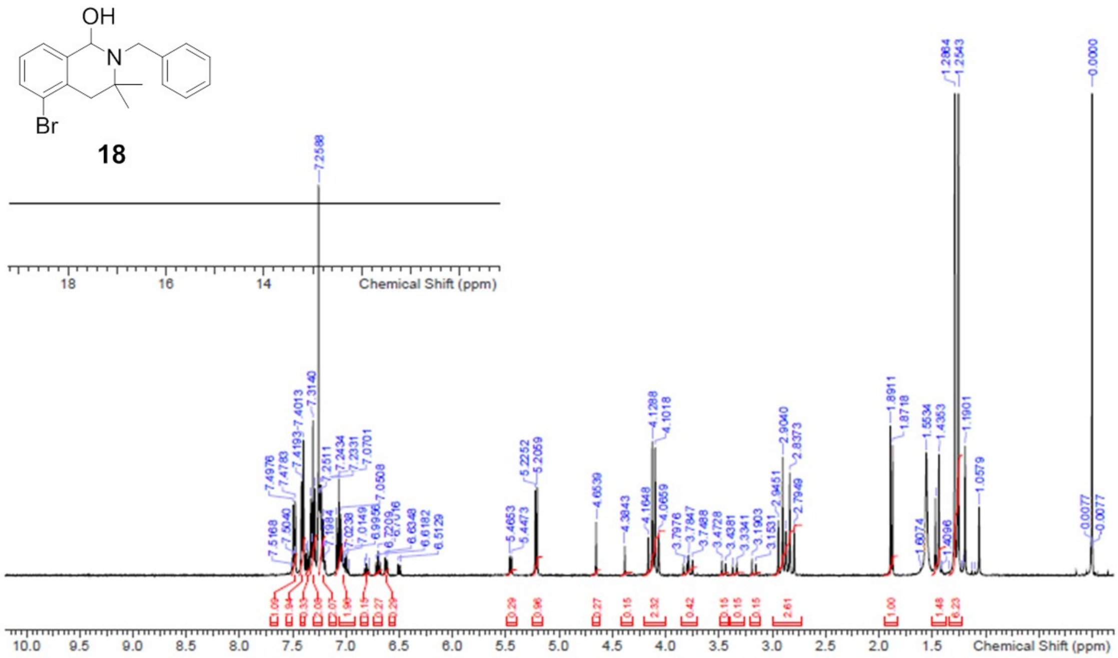

Supplement: RA-008-C7RA13627G-s001 [file RA-008-C7RA13627G-s001.pdf]
